# Management of respiratory distress in moderate and late preterm infants: clinical trajectories in the Neobs study

**DOI:** 10.1007/s00431-023-05259-8

**Published:** 2023-10-12

**Authors:** Isabelle Guellec, Thierry Debillon, Cyril Flamant, Pierre-Henri Jarreau, Benjamin Serraz, Pierre Tourneux

**Affiliations:** 1 Neonatal Intensive Care Unit, University Hospital of Nice-Côte d’Azur, 06200 Nice, France; 2 Neonatology Intensive Care Unit, University Hospital of Grenoble, Grenoble, France; 3 Neonatal Intensive Care Unit, University Hospital of Nantes, Nantes, France; 4 Neonatal Intensive Care Unit of Port-Royal, AP–HP Centre–Université de Paris, Paris, France; 5 Medical Affairs, Chiesi SAS, Bois Colombes, France; 6 Neonatal Intensive Care Unit, University Hospital of Amiens, University of Picardy Jules Verne, Amiens, France

**Keywords:** Clinical trajectory, Non-invasive ventilation, Preterm infants, Respiratory distress, Surfactant

## Abstract

Management of respiratory distress (RD) in the extremely preterm newborn meets recommendations. Few data are available concerning the management and the clinical course of moderate and late preterms with RD. Clinical course and management among moderate (30–33 weeks (wks) of gestation) and late preterms (34–36 wks) were assessed in the Neobs study, a French neonatal observational cohort study (2018) of preterms with RD in the first 24 h of life. Clinical course was defined as stable (use of non-invasive ventilation (NIV) only), initially severe (initial use of invasive ventilation (IV)), and worsening (switch off IV after NIV support). Surfactant therapy instillation and withdrawal of all ventilator support at 72 h were recorded. Among moderate (*n* = 279) and late (*n* = 281) preterms, the clinical course was similar (*p* < 0.27): stable (82.1 and 86.8%), worsening (11.8% and 9.3%), and initially severe RD (6.1% and 3.9%), respectively. Surfactant was administered more frequently in the moderate versus late preterm groups (28.3% vs 16.7%; *p* < 0.001). The recommended surfactant dose (200 mg/kg) was administered in 53.3–83.3% of moderate and 42.1–63.2% of late preterms according to the clinical course. Withdrawal of ventilatory support at 72 h was observed in 40.0% and 70.0% of moderate and late preterms, respectively (*p* < 0.05), and was significantly (*p* < 0.001) associated with clinical course (the minus proportion among the worsening group).

*Conclusion*: While the proportion of clinical course pattern is similar in moderate and late preterm infants, the management of RD varies with gestational age, with late preterm infants being managed later in life and moderate premature infants weaned from ventilation at a later stage.

**Table:** 

**What is Known:** *• There is a lack of clear guidance on the management of respiratory distress (RD) in moderate-to-late preterm infants.* *• Neobs was a multicentre, observational study designed to characterise the real-world management of moderate-to-late preterm infants with RD in France.*	
**What is New:** *• Secondary analyses of Neobs study data found that ventilatory support strategies were dependent on gestational age despite a similar clinical course.* *• At 30–33 weeks of gestation (wks), infants were more likely to receive non-invasive ventilation at delivery, while 34–36 wks infants were more likely to be managed using a wait-and-see approach.*

**What is Known:**

*• There is a lack of clear guidance on the management of respiratory distress (RD) in moderate-to-late preterm infants.*

*• Neobs was a multicentre, observational study designed to characterise the real-world management of moderate-to-late preterm infants with RD in France.*

**What is New:**

*• Secondary analyses of Neobs study data found that ventilatory support strategies were dependent on gestational age despite a similar clinical course.*

*• At 30–33 weeks of gestation (wks), infants were more likely to receive non-invasive ventilation at delivery, while 34–36 wks infants were more likely to be managed using a wait-and-see approach.*

## Introduction

Respiratory distress (RD) in moderate-to-late preterm infants occurs in up to 20% of those born at 32–34 weeks of gestation (wks) and in up to 8% of those born at 34–36 wks [1–3]. Contrary to the situation for preterm infants born at < 32 wks, little is known about the clinical course of RD, or the use and duration of ventilatory support needed, in moderate and late preterm infants (30–33 wks and 34–36 wks, respectively). 

Current guidelines include little specific guidance on the management of RD, including the use of ventilatory support, in moderate-to-late preterm infants [4]. As a result, clinicians must choose between treating these newborns aggressively using the model developed for very preterm infants, which carries the risk of iatrogenicity or adopting a wait-and-see approach, which carries the risk of worsening RD and a switch from non-invasive to invasive ventilation.

Neobs (derived from NEonatal OBservational Study) was a large observational study designed to characterise the respiratory care provided to moderate-to-late preterm infants in France [5]. Debillon et al. previously reported that in the Neobs study, continuous positive airway pressure (CPAP) was widely used in the delivery room and that the less-invasive surfactant administration method was chosen for 34.4% of the surfactant administrations for the management of respiratory distress in moderate-to-late preterm infants. We hypothesised that the management of neonates presenting with respiratory distress was more related to their term than to the evolution of the disease, and that the least immature neonates were more frequently managed by a wait-and-see strategy.

Herein, this analysis of Neobs study data aimed to investigate discrepancies between clinical trajectories and management practice according to gestational age in moderate-to-late preterm infants with RD and determine factors associated with the clinical course.

## Materials and methods

### Study design

The multicentre, prospective, observational Neobs study was conducted in 46 maternity units (with neonatal intensive-care or neonatal resuscitation unit) caring for preterm infants in France [5].

### Study population

Preterm infants born between 30 + ^0/7^ and 36 + ^6/7^ wks who had early neonatal respiratory failure, defined as the need for non-invasive ventilation (NIV, i.e., high-flow nasal cannula (HFNC), CPAP, or nasal intermittent positive pressure ventilation (NIPPV)) or invasive mechanical ventilation (conventional or high frequency) during the first 24 h after birth and who were hospitalised in the first 24 h of life, were eligible for enrolment. Infants were excluded if they had received ventilatory support for a non-respiratory disease or malformation disorder, or if they died within the first 24 h of life.

### Data collection

Neonates were enrolled from February 9th, and October 31st, 2018, and data were collected until their return home or their 60th day of life. For each included infant, the following data were recorded by the investigator and his/her team at each inclusion or follow-up visit using an electronic Case Report Form. The timepoints of interest were at 24 h, a follow-up visit at 72 h, at day 7, and at hospital discharge or on day 60 (if the infant was still in hospital). As Neobs was an observational study, the practitioner in charge of the newborn had not received any specific management instructions.

Type of pathology (respiratory distress syndrome due to primary surfactant deficiency (RDS) or transient tachypnoea of the newborn (TTN)) was determined by the practitioner in charge at 24 h of life.

### Outcomes

The study population was stratified into two groups based on gestational age at delivery (30–33 and 34–36 wks). For each group, the main outcome was the clinical course of infants during the first 7 days of life, with clinical course classified as stable, worsening, or initially severe RD based on the need for ventilatory support. Infants with stable RD were those managed with NIV only (i.e., HFNC, CPAP, or NIPPV) until the withdrawal of ventilatory support; infants with worsening RD were initially managed with NIV and required invasive mechanical ventilation during the first 7 days of life; and infants with initially severe RD required invasive mechanical ventilation before any other method of respiratory support.

Secondary outcomes explored in this analysis included surfactant use and time to withdrawal of ventilatory support. Surfactant therapy used in France was poractant alpha and the dosages recommended in France were 200 mg/kg and 100 mg/kg for the first and second instillations, respectively and time to withdrawal of ventilatory support. Secondary outcomes were assessed by gestational age (30–33 and 34–36 wks) and clinical course (stable, worsening, and initially severe RD).

### Statistical analysis

Neobs was a descriptive study of ventilatory practices used for preterm infants > 30 weeks GA in France in 2018. These practices were therefore described taking several criteria into account (proportion of infants having received NIV at H24, H72, time to introduce NIV, respiratory support before CPAP, description of NIV use, proportion of infants having been intubated at H24, etc.). However, no available clinical practice data existed in 2018 to anticipate the true proportions for each of these parameters. Thus, sample size was calculated based on the precision of the confidence interval (CI) estimate for the proportions for the different endpoints in each stratum. The sample size was dependent upon the percentage expected, the necessary precision, and the alpha risk. We used the least favourable hypothesis with a proportion of 50% of infants requiring mechanical ventilation, a two-sided 95% CI, and ± 6% precision, resulting in a sample size of 270 infants per stratum (i.e., 540 infants were required for inclusion in this study).

Qualitative variables were presented as the number and percentage of patients, and quantitative variables as mean and standard deviation (SD), or median and range. Quantitative variables were compared using the Student’s *t*-test or the Wilcoxon–Mann–Whitney test according to distribution and homogeneity of variance. Categorical variables were assessed using the Pearson’s chi-square test or the Fisher’s exact test. All statistical tests were two-sided, and the significance level was set at *p* < 0.05.

Logistic regression models were used to identify the type of pathology and surfactant therapy-related factors associated with worsening versus stable clinical course. Each potential explanatory variable was tested by univariate analysis, and variables with *p* < 0.25 (conservative threshold) were then included in a multivariate logistic regression model with stepwise selection of the adjusted variables (i.e., elimination of variables with *p* > 0.05). Analyses were performed using available data, with no imputation of missing values. SAS® software (version 9.4, SAS Institute, NC, USA) was used for all analyses.

### Legal information—ethics

This study was performed in line with the principles of the Declaration of Helsinki. The Neobs study received ethical approval from the West V Rennes Research Ethics Committee (Comités de Protection des Personnes, CPP) in October 2017, and from the French Data Protection Authority (Commission Nationale de l’Informatique et des Libertés, CNIL) on 13 July, 2017. Parents or legal guardians provided oral informed consent for participation in the study.

## Results

### Study population

The Neobs study included 575 infants, 560 of whom had complete data available for analysis. The analysis population comprised 279 infants born at 30–33 wks and 281 infants born at 34–36 wks. Full baseline characteristics relating to the mother, gestation period, and neonate groups have been reported previously [5]. Aetiology of respiratory distress was significantly different among categories of gestational age. RDS was observed in 138/265 (49.5%) of moderate and 88/281 (30.6%) of late preterms, respectively, and TTN was observed in 127/279 (45.5%) and 184/281 (65.5%) of moderate and late preterms, respectively (*p* < 0.001).

### Pregnancy and neonatal characteristics by clinical course

Stable RD was observed in 229/279 (82.1%) of moderate and 244/281 (86.8%) of late preterms, worsening RD in 33/279 (11.8%) of moderate and 26/281 (9.3%) of late preterms, and initially severe RD among 17/279 (6.1%) of moderate and 11/281 (3.9%) of late preterms. There were no statistical differences in the frequency of clinical course status between the moderate and late preterm groups (*p* = 0.27).

The use of antenatal corticosteroids was significantly associated with clinical course in the 30–33 wks group (Table 1). In 30–33 wks infants exposed to antenatal corticosteroids, the proportion with stable RD was significantly higher than in non-exposed infants (*p* = 0.01). For both moderate and late preterm infants, an Apgar score < 7 at 5 min (*p* < 0.01 and *p* = 0.02, respectively) and having a haemodynamic complication (*p* < 0.01 in both groups) were each associated with worsening or initially severe clinical course. Worsening or initially severe RD was significantly less common among multiple births in the 30–33 wks group (*p* < 0.01) but not in the 34–36 wks group (*p* = 0.07).

**Table 1:** Pregnancy and neonatal characteristics by clinical course subgroup

	**30–33 wks infants (*n* = 279)**				**34–36 wks infants (*n* = 281)**			
	**Stable RD** **(*n* = 229)**	**Worsening RD** **(*n* = 33)**	**Initially severe RD (*n* = 17)**	***p***	**Stable RD** **(*n* = 244)**	**Worsening RD** **(*n* = 26)**	**Initially severe RD (*n* = 11)**	***p***
**Pregnancy type, *n* (%)**								
Singleton	135 (79.7)	26 (14.8)	15 (8.5)	< 0.01	153 (83.6)	20 (10.9)	10 (5.5)	0.07
Multiple	94 (91.3)	7 (6.8)	2 (1.9)		91 (92.9)	6 (6.1)	1 (1.0)	
**Gestational age, *n* (%)**								
30 wks	56 (78.9)	8 (11.3)	7 (9.9)	0.20	–	–	–	
31 wks	58 (81.7)	7 (9.9)	6 (8.5)		–	–	–	
32 wks	64 (79.0)	14 (17.3)	3 (3.7)		–	–	–	
33 wks	51 (91.1)	4 (7.1)	1 (1.8)		–	–	–	
34 wks	–	–	–		116 (82.9)	15 (10.7)	9 (6.4)	0.21
35 wks	–	–	–		79 (90.8)	6 (6.9)	2 (2.3)	
36 wks	–	–	–		49 (90.7)	5 (9.3)	0 (0)	
**Antenatal corticosteroid use, *n* (%)**								
Yes	185 (87.7)	20 (9.5)	6 (2.8)	0.01	98 (85.2)	12 (10.4)	5 (4.4)	0.75
No	43 (65.2)	12 (18.2)	11 (16.7)		145 (88.4)	13 (7.9)	6 (3.7)	
**Apgar score at 5 min, *n* (%)**								
≥ 7	216 (85.7)	27 (10.6)	11 (4.3)	< 0.01	224 (87.8)	24 (9.4)	7 (2.8)	0.02
< 7	12 (54.6)	5 (22.7)	5 (22.7)		18 (75.0)	2 (8.3)	4 (16.7)	
**Haemodynamic complication, *n* (%)**								
Yes	17 (68.0)	8 (32.0)	0	< 0.01	3 (33.3)	4 (44.4)	2 (22.2)	< 0.01
No	212 (83.5)	25 (9.8)	17 (6.7)		241 (88.6)	22 (8.1)	9 (3.3)	
**Type of pathology**								
Hyaline membrane disease	98 (71.0%)	29 (21.0%)	11 (8.0%)		61 (69.3%)	21 (23.9%)	6 (6.8%)	
Transient tachypnoea of the newborn	122 (96.1%)	2 (1.6%)	3 (2.4%)	< 0.01	176 (95.7%)	3 (1.6%)	5 (2.7%)	< 0.01
**Mean FiO**_**2**_** maximum during hospitalisation**	39 (± 21.3)	59.8 (± 25.5)	66.5 (± 29)	< 0.01	36.2 (± 19.8)	54.9 (± 24.7)	62.1 (± 33.6)	< 0.01

Analyses were performed using available data, with no imputation of missing values

*FiO*_*2*_ fraction of inspired oxygen, *RD* respiratory distress, *wks* weeks of gestation

Type of pathology was significantly different according to clinical course both among moderate and late preterm infants (*p* < 0.0001). TTN was considered in the stable clinical course for 122/127 (96.1%) and 176/184 (95.7%) of moderate and late preterm infants, respectively. In a multivariate analysis using stable clinical course as the reference, which was adjusted for type of pregnancy, Apgar score at 5 min, and the presence of ≥ 1 haemodynamic complication, moderate preterms with both worsening RD and initially severe RD were more likely to be diagnosed as RDS than TTN, while among late preterms, this association remained significantly only with the worsening clinical course group (Table 2).

**Table 2:** Regression analysis for factors associated with clinical course

	**30–33 wks**				**34–36 wks**			
	**Population with worsening RD vs stable RD**		**Population with initially severe RD vs stable RD**		**Population with worsening RD vs stable RD**		**Population with initially severe RD vs stable RD**	
	**OR (95% CI)**	***p***	**OR (95% CI)**	***p***	**OR (95% CI)**	***p***	**OR (95% CI)**	***p***
**Unadjusted model**								
Type of pathology (respiratory distress syndrome vs transient tachypnoea of the newborn)	18.05 (4.20–77.52)	< 0.01	4.56 (1.24–16.82)	< 0.01	20.19 (5.82–70.04)	< 0.01	3.46 (1.02–11.75)	0.046
**Adjusted model**								
Type of pathology (respiratory distress syndrome vs transient tachypnea of the newborn)	15.94 (3.63–70.00)	< 0.01	5.16 (1.53–19.63)	0.02	19.10 (5.41–67.45)	< 0.01	3.02 (0.80–11.37)	0.10
Type of pregnancy (multiple vs singleton)	0.32 (0.12–0.83)	0.02	0.23 (0.05–1.10)	0.06	0.52 (0.18–1.54)	0.24	0.07 (0.01–0.84)	0.04
Apgar score at M5 (< 7 vs ≥ 7)	2.68 (0.71–10.06)	0.15	5.71 (1.44–22.59)	0.03	1.05 (0.18–6.08)	0.96	9.41 (2.18–40.67)	< 0.01
At least 1 haemodynamic complication	3.44 (1.20–9.87)	0.02	< 0.01 (< 0.01– > 999.9)	0.07	13.31 (1.96–90.30)	< 0.01	25.67 (2.75–239.78)	< 0.01

*CI* confidence interval, *RD* respiratory distress, *wks* weeks of gestation

### Ventilatory support by clinical course

**Fig. 1:**
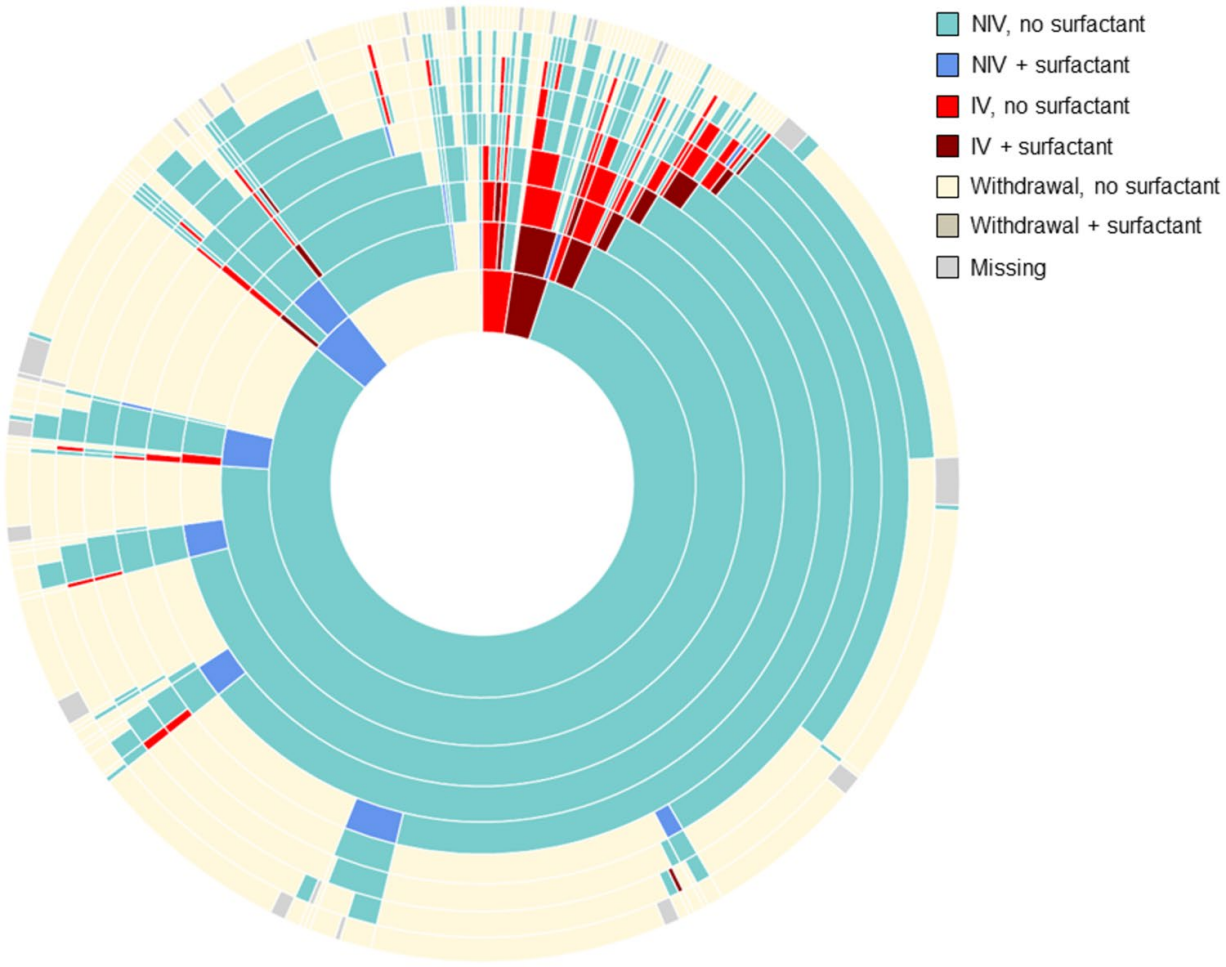
Sunburst chart depicting patterns of ventilatory support over time in moderate-to-late preterm infants in the Neobs study (*n* = 560). Each ring of the sunburst equates to a time since birth, with the inner ring being 0 h since birth, and subsequent rings (from inside to outer) being 3 h, 6 h, 12 h, 24 h, 72 h, 7 days, and 60 days since birth, respectively. An interactive version of this figure is available at https://www.chiesi.fr/documenti/749_sunburst_statuts_ventilatoires_h0_d60_mar2023.html. *CPAP* continuous positive airway pressure, *IV* invasive ventilation, *HFNC* high-flow nasal cannula, *NIPPV* nasal intermittent positive pressure ventilation, *NIV* non-invasive ventilation (HFNC + CPAP + NIPPV)

An overview of the ventilatory support received by all infants during the first 60 days of life is presented in Fig. 1. An interactive version of this figure is available at https://www.chiesi.fr/documenti/749_sunburst_statuts_ventilatoires_h0_d60_mar2023.html.

**Fig. 2:**
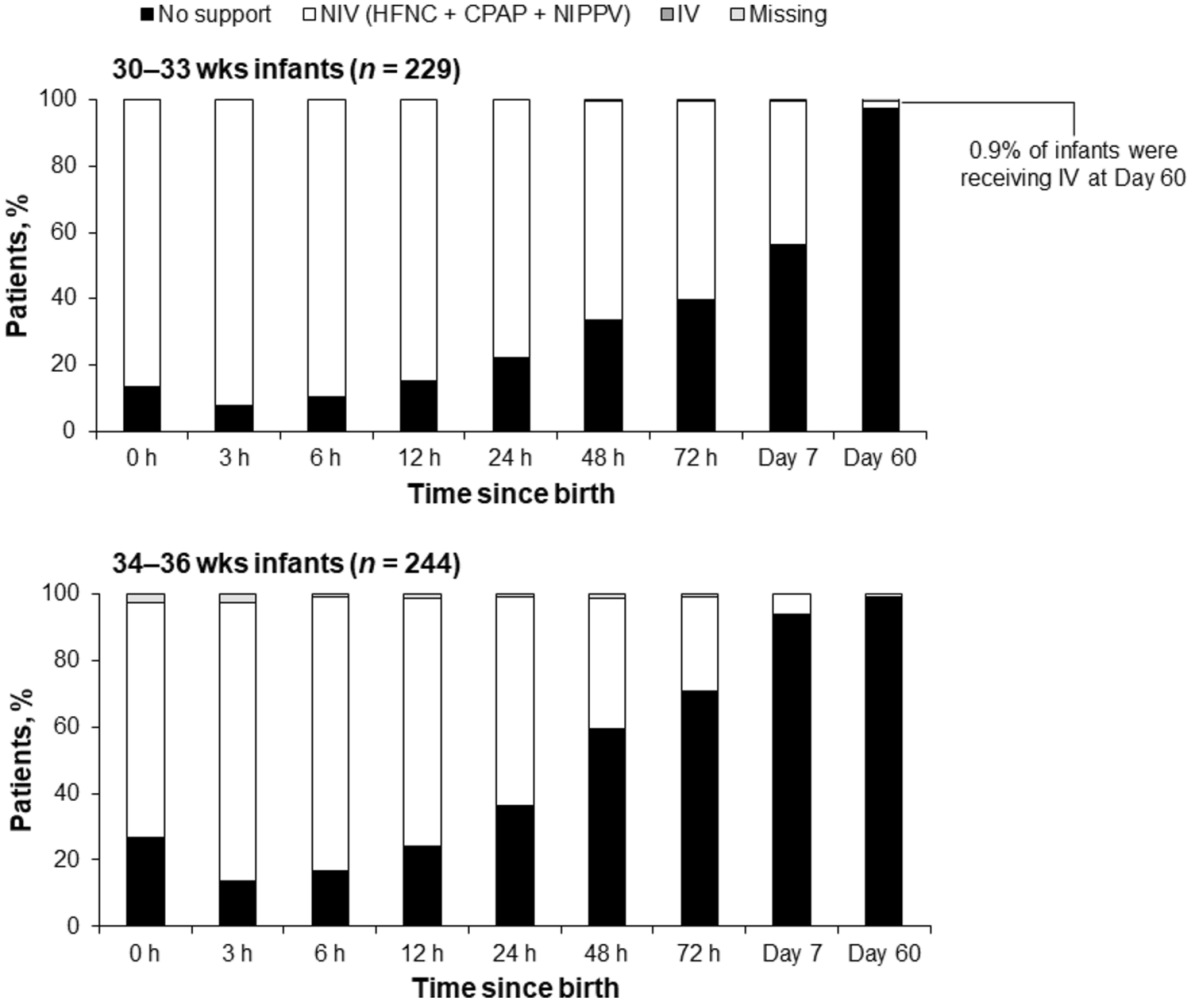
Patterns of ventilatory support over time in 30–33 wks and 34–36 wks infants with stable respiratory distress. Analyses were performed using available data, with no imputation of missing values. In the 30–33 wks group, 0.9% of infants were receiving IV at Day 60; no other 30–33 wks or 34–36 wks infants with stable respiratory distress received IV at any time point. *CPAP* continuous positive airway pressure, *HFNC* high-flow nasal cannula, *IV* invasive ventilation, *NIPPV* nasal intermittent positive pressure ventilation, *NIV* non-invasive ventilation, *wks* weeks of gestation

Among preterm infants with stable RD, 86.4% of the 30–33 wks group and 70.5% of the 34–36 wks group received NIV at birth, which increased to 92.1% and 83.6%, respectively, at 3 h after birth (Fig. 2). Support with NIV used HFNC in 5.2% and 15.6% of the 30–33 wks group and 34–36 wks group, respectively, at 3 h after birth. At 72 h, 39.7% of the 30–33 wks group and 70.5% of the 34–36 wks group were weaned off ventilation.

**Fig. 3:**
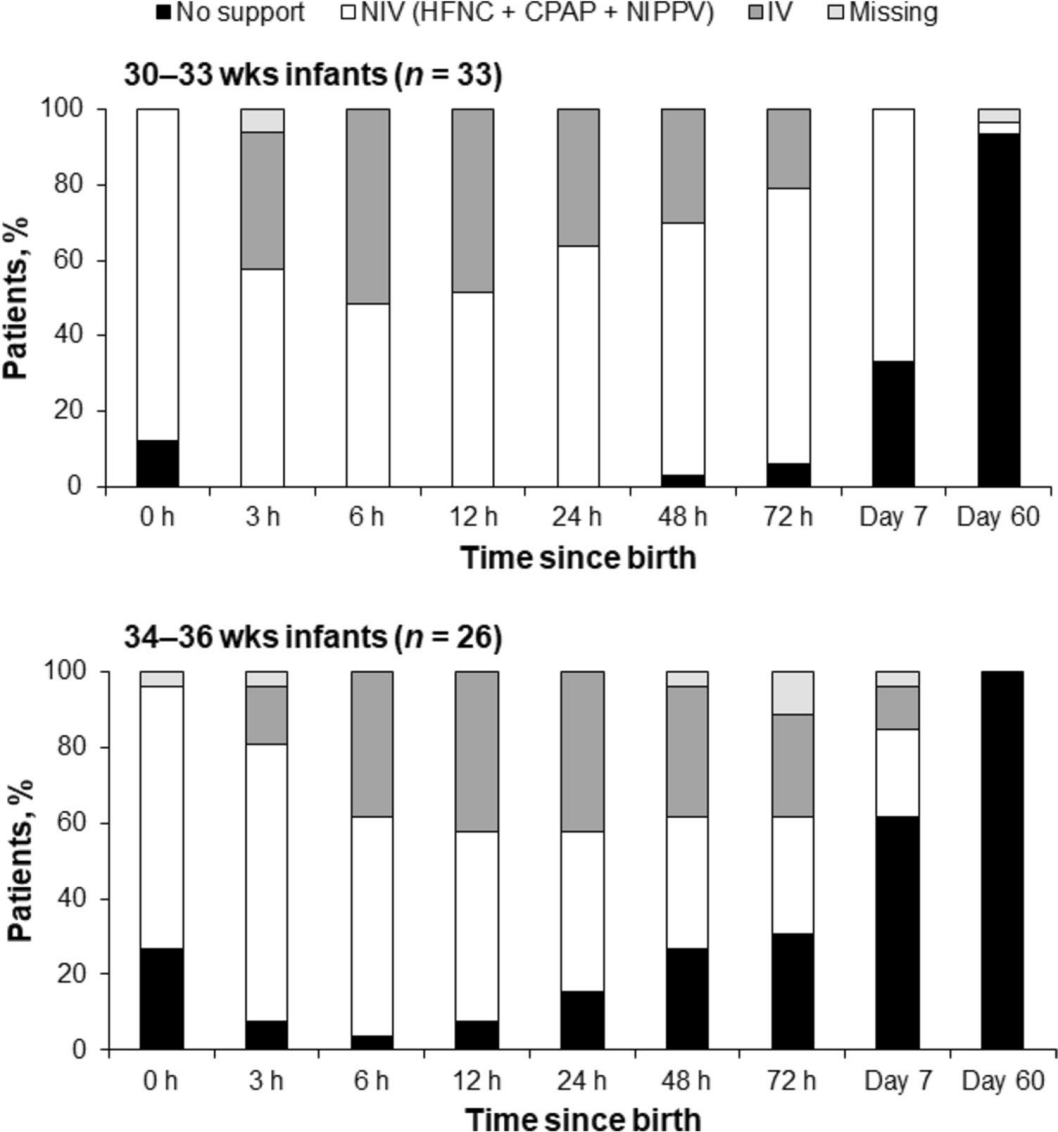
Patterns of ventilatory support over time in 30–33 wks and 34–36 wks infants with worsening respiratory distress. Analyses were performed using available data, with no imputation of missing values. *CPAP* continuous positive airway pressure, *HFNC* high-flow nasal cannula, *IV* invasive ventilation, *NIPPV* nasal intermittent positive pressure ventilation, *NIV* non-invasive ventilation, *wks* weeks of gestation

In infants with worsening RD, the time period in which the highest proportion were receiving mechanical ventilation was 6–12 h after birth for the 30–33 wks group and 12–24 h after birth for the 34–36 wks group (Fig. 3). At birth, 12.1% of the 30–33 wks group and 26.9% of the 34–36 wks group received no ventilatory support, while 87.9% and 69.2%, respectively, were supported by NIV (including 18.2% and 15.4% on HFNC, respectively). At 72 h, 21.2% and 26.9% of the 30–33 wks group and 34–36 wks group remained on mechanical ventilation, respectively, and 6.1% and 30.8% of infants, respectively, had been weaned off ventilatory support.

**Fig. 4:**
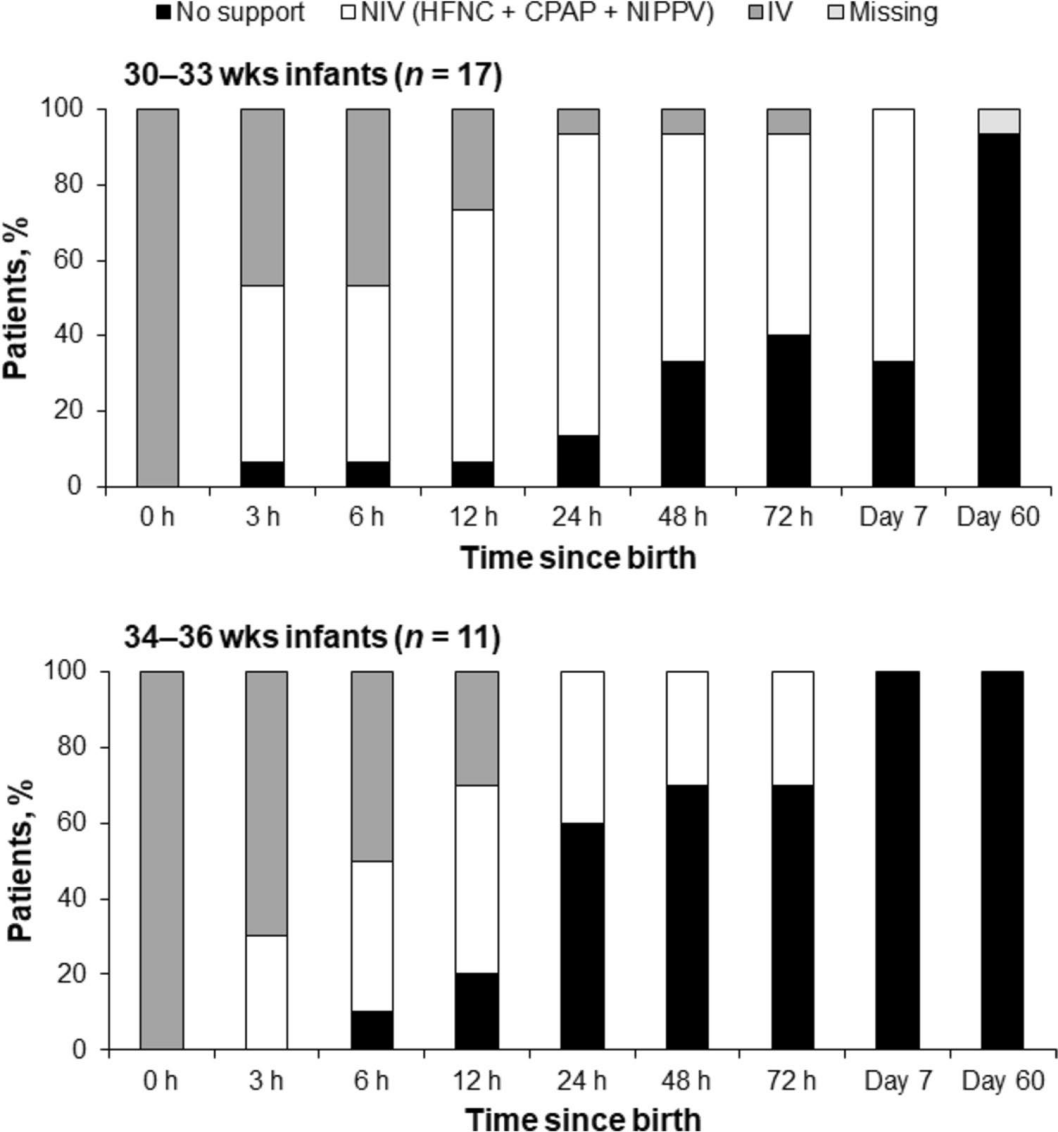
Patterns of ventilatory support over time in 30–33 wks and 34–36 wks infants with initially severe respiratory distress. Analyses were performed using available data, with no imputation of missing values. *CPAP* continuous positive airway pressure, *HFNC* high-flow nasal cannula, *IV* invasive ventilation, *NIPPV* nasal intermittent positive pressure ventilation, *NIV* non-invasive ventilation, *wks* weeks of gestation

In infants with initially severe RD, after receiving mechanical ventilation at birth, 93.3% of the 30–33 wks group and 100% of the 34–36 wks group were weaned from mechanical ventilation at 24 h of life (Fig. 4). At 24 h, NIV was received by 80.0% of the 30–33 wks group and 40.0% of the 34–36 wks group. Withdrawal of all ventilatory support at 72 h was observed in 40.0% and 70.0% of the 30–33 wks and 34–36 wks groups, respectively.

### Surfactant therapy by clinical course

Overall, surfactant therapy was used in 79/279 (28.3%) of 30–33 wks infants and 47/281 (16.7%) of 34–36 wks infants (*p* < 0.001 for comparisons between clinical course subgroups; Table 3). For both 30–33 wks and 34–36 wks groups, the time from birth to first surfactant administration was significantly different between patients with stable, worsening, or initially severe RD (*p* = 0.03 and *p* = 0.02, respectively). In the 30–33 wks group, the mean ± SD time from birth to first surfactant administration was longest for those with worsening clinical course (9.4 ± 13.2 h), whereas in the 34–36 wks group, it was longest for those with stable and worsening RD (14.7 ± 11.5 and 14.5 ± 19.9 h, respectively). The recommended surfactant dose (200 mg/kg) was administered in 53.3–83.3% of 30–33 wks infants and 42.1–63.2% of 34–36 wks infants across clinical course subgroups; 16.7–33.3% of the 30–33 wks group and 21.1–57.1% of the 34–36 wks group received less than the recommended dose. In the multivariate model, following adjustment for both preterm groups, surfactant therapy was significantly more likely to be used in the worsening and the initially severe RD clinical course (Table 4).

**Table 3:** Surfactant administration by clinical course subgroup

	**30–33 wks infants (*n* = 279)**				**34–36 wks infants (*n* = 281)**			
	**Stable RD** **(*n* = 229)**	**Worsening RD** **(*n* = 33)**	**Initially severe RD** **(*n* = 17)**	***p***	**Stable RD** **(*n* = 244)**	**Worsening RD** **(*n* = 26)**	**Initially severe RD** **(*n* = 11)**	***p***
**Surfactant administration during hospitalisation, *n* (%)**								
Yes	38 (48.1)	32 (40.5)	9 (11.4)	< 0.0001	20 (42.6)	20 (42.6)	7 (14.9)	< 0.001
No	191 (95.5)	1 (0.5)	8 (4.0)		224 (95.7)	6 (2.6)	4 (1.7)	
**Total number of surfactant administrations, *n* (%)**								
0	191 (83.4)	1 (3.1)	8 (47.1)	< 0.0001	224 (91.8)	6 (23.1)	4 (36.4)	< 0.01
1	37 (16.2)	27 (84.4)	7 (41.2)		20 (8.2)	17 (65.4)	7 (63.6)	
≥ 2	1 (0.4)	4 (12.5)	2 (11.8)		0	3 (11.5)	0	
**Surfactant dose, *n* (%)**								
< 200 mg/kg	9 (25.0)	10 (33.3)	1 (16.7)	0.79	4 (21.1)	9 (47.4)	4 (57.1)	0.32
200 mg/kg	21 (58.3)	16 (53.3)	5 (83.3)		12 (63.2)	8 (42.1)	3 (42.9)	
> 200 mg/kg	6 (16.7)	4 (13.3)	0		3 (15.8)	2 (10.5)	0	
**Time from birth to first surfactant administration (h), mean ± SD**	5.9 ± 6.7	9.4 ± 13.2	2.9 ± 6.2	0.03	14.7 ± 11.5	14.5 ± 19.9	2.6 ± 2.3	0.02

Analyses were performed using available data, with no imputation of missing values

*RD* respiratory distress, *SD* standard deviation, *wks* weeks of gestation

**Table 4:** Regression analysis for surfactant administration and clinical course

	**30–33 wks**				**34–36 wks**			
	**Population with worsening RD vs stable RD**		**Population with initially severe RD vs stable RD**		**Population with worsening RD vs stable RD**		**Population with initially severe RD vs stable RD**	
	**OR (95% CI)**	***p***	**OR (95% CI)**	***p***	**OR (95% CI)**	***p***	**OR (95% CI)**	***p***
**Unadjusted model**								
Surfactant during hospitalisation	160.84 (21.32– > 999.99)	< 0.01	19.60 (5.28–72.71)	< 0.01	37.33 (13.46–103.60)	< 0.01	19.60 (5.28–72.71)	< 0.01
**Adjusted model**								
Surfactant during hospitalisation	150.27 (19.55– > 999.99)	< 0.001	6.94 (2.25–21.41)	< 0.01	41.93 (14.21–123.67)	< 0.01	20.61 (4.79–88.68)	< 0.01
Type of pregnancy (Multiple vs Singleton)	0.30 (0.10–0.86)	0.03	0.20 (0.04–0.95)	0.04	0.55 (0.17–1.79)	0.32	0.11 (0.01–1.36)	0.09
Apgar score at M5 (< 7 vs ≥ 7)	2.26 (0.44–9.10)	0.27	5.60 (1.46–21.55)	0.01	1.00 (0.17–6.05)	1.00	7.21 (1.43–36.22)	0.02
At least 1 haemodynamic complication	2.20 (0.63–7.71)	0.22	< 0.01 (< 0.001– > 999.99)	0.97	21.28 (2.721–166.51)	0.01	35.60 (3.50–61.63)	< 0.01

*CI* confidence interval, *RD* respiratory distress, *wks* weeks of gestation

### Withdrawal of ventilatory support by clinical course

The proportion of infants withdrawn from ventilatory support at 72 h and 7 days was significantly associated with gestational age and a long clinical course (Table 5). At 72 h, 90/270 (33.3%) of the moderate preterm group and 183/280 (65.4%) of the late preterm group were weaned off ventilation (*p* < 0.001) and at 7 days, 143/270 (53%) and 255/280 (91.1%) at 7 days. When infants were stratified by gestational age, differences in weaning ventilation between clinical course subgroups were significant for both 30–33 wks (*p* < 0.0001 and *p* = 0.03 at 72 h and 7 days, respectively) and 34–36 wks groups (*p* < 0.0001 at both time points).

**Table 5:** Withdrawal of ventilatory support by clinical course subgroup

**Withdrawal of ventilatory support**										
		**Population 30 + 0–33 + 6 wks**				**Population 34 + 0–36 + 6 wks**				**Overall *p***
**Within the first 72 h, *n* (%)**	Yes	90 (33.3)				183 (65.4)				< 0.001
	No	180 (66.7)				97 (34.6)				
		Stable RD (*n* = 229)	Worsening RD (*n* = 33)	Initially severe (*n* = 17)	***p***	Stable RD (*n* = 244)	Worsening RD (*n* = 26)	Initially severe (*n* = 11)	***p***	
	Yes	83 (37.4)	1 (3.1)	6 (37.5)	< 0.001	169 (69.5)	7 (26.9)	7 (63.6)	< 0.0001	
	No	139 (62.6)	31 (96.9)	10 (62.5)		74 (30.5)	19 (73.1)	4 (36.4)		
**Within the first 7 days, *n* (%)**	Yes	143 (53%)				255 (91.1%)				< 0.001
	No	127 (47%)				25 (8.9%)				
		Stable RD (*n* = 229)	Worsening RD (*n* = 33)	Initially severe (*n* = 17)		Stable RD (*n* = 244)	Worsening RD (*n* = 26)	Initially severe (*n* = 11)	***p***	
	Yes	126 (56.8)	11 (34.4)	6 (37.5)	0.03	229 (94.2)	16 (61.5)	10 (90.9)	< 0.0001	
	No	96 (43.2)	21 (65.6)	10 (62.5)		14 (5.8)	10 (38.5)	1 (9.1)		

Analyses were performed using available data, with no imputation of missing values

*Q* quartile, *RD* respiratory distress, *wks* weeks of gestation

## Discussion

This descriptive analysis of data from the Neobs study showed that ventilatory support strategies in moderate-to-late preterm infants with RD were dependent on gestational age. In 30–33 wks infants, use of NIV in the delivery room was common and often prolonged, with 46–67% of infants remaining on NIV at 72 h. In contrast, delivery room use of NIV was less common in 34–36 wks infants, who were more likely to be managed using a wait-and-see strategy. The latter approach was utilised in almost 50% of 34–36 wks infants with worsening RD. Indeed, it should be noted that during this progression towards worsening RD, a large proportion of newborns, in particular those who were considered more mature (34–36 wks), were not managed with NIV at the time of birth. This agrees with a recent meta-analysis, which could not recommend the use of CPAP in late preterm infants at birth due to insufficient evidence [6]. The question remains open as to whether it is possible to anticipate an aggravation in these patients, in order to administer ventilatory support as soon as possible. In this study, the duration of ventilatory support was greater among 34–36 wks infants with worsening RD than in those with stable or initially severe RD. Our study and the recent meta-analysis [6] collectively highlight the need for prospective, randomised studies to evaluate the utility of NIV in the delivery room for late preterm infants with RD.

Among the infants who were stable on NIV, one question arises—whether being stabilised with early NIV made it possible to limit the evolution towards an unfavourable outcome. This observational study does not allow us to answer this question formally, but studies have demonstrated that early CPAP stabilises infants with transient tachypnoea of the newborn and reduces the duration and severity of RD [7, 8]. In addition, we observed that the duration of NIV was dependent on the gestational age, which raises the question of the needs of the newborn versus the practices of the service. Teams have proposed algorithms for weaning infants off NIV [9], but no clear guidelines are yet available.

The results for infants with initially severe RD should be interpreted with caution due to the low number of patients in this subgroup. However, in this population, it is important to highlight the rapidity with which neonates were weaned from mechanical ventilation in the first day of life, raising the issue of overtreatment in this population. The apparent reluctance of neonatologists to mechanically ventilate patients in the birth room is to be counterbalanced with these results, and those of a recent study suggesting that invasive mechanical ventilation at birth does not adversely affect lung function in later childhood in a population of moderate preterm newborns (with a mean gestational age of 30 wks) and a short duration of mechanical ventilation [10]. Of note, it is unlikely that this result can be extrapolated to all premature newborns who may present with more severe respiratory illnesses.

In this study, infants with worsening or initially severe RD were frequently administered surfactant therapy. This finding is likely due to indication bias; those who required surfactant had more severe RD and were more likely to show worsening over time. Overall, surfactant use was relatively uncommon (only 17–28% of cases depending on gestational age). Current recommendations state that the threshold for surfactant administration is when the fraction of inspired oxygen (FiO_2_) is > 30%, with a CPAP of ≥ 6 cm H_2_O [4]. In the Neobs study, the median FiO_2_ at the time of surfactant administration was 40% (interquartile range, 34.5–50%) [5], though it should be noted that clinical guidelines during the study period recommended a FiO_2_ threshold of 30–40% [11]. The study data do not provide any further insight into why clinicians waited until higher maximum FiO_2_ thresholds were reached before starting surfactant. Given the lack of data comparing outcomes after surfactant therapy at different FiO_2_ thresholds and during different modes of ventilatory support, the ideal threshold for surfactant therapy in late preterm infants is yet to be determined [12]. However, higher FiO_2_ at the time of surfactant administration has been shown to significantly increase the risk of mechanical ventilation prior to 72 h of life [13]. In the current study, surfactant therapy was also delayed, particularly in 34–36 wks infants (approximately 15 h after birth in those with stable or worsening RD), and surfactant was rarely given at the recommended dose. This finding is in line with other observational studies carried out previously in Europe, which identified similar proportions of surfactant use at non-recommended doses [14, 15], and questions the efficacy of surfactant and the inherent reasons for delayed use. Current hypotheses suggest a problem with packaging [16] or erroneous rounding of doses. This also highlights the problem of generalizing the results obtained in these observational studies within our Discussion section.

An important question from a clinical perspective is whether delaying surfactant administration has an impact on clinical trajectory in 30–36 wks preterm infants with RD. After adjustment for potential confounders, the odds ratio value for worsening RD in 30–33 wks infants decreased but did not indicate any significant benefit. However, this may be due to a lack of study power given the low number of patients in some subgroups. It has previously been reported that early surfactant administration in < 34 wks preterm infants receiving ventilatory support decreases the risk of pulmonary injury, neonatal death, and chronic lung disease compared with delaying surfactant administration until RD worsens [17]. One final issue to note regarding the use of surfactant is that, while clinicians agree that the initial dose should be 200 mg/kg [18], we found that surfactant was initiated at the recommended dose in only 53.3–83.3% of 30–33 wks infants and 42.1–63.2% of 34–36 wks infants across clinical course subgroups. Moreover, 16.7–33.3% and 21.1–57.1% of the 30–33 wks and 34–36 wks infants, respectively, received less than the recommended surfactant dose.

A key strength of this study is that it provides some of the first data on clinical trajectories in moderate-to-late preterm infants with RD. However, several limitations should be considered when interpreting these findings. As previously described, most infants included in the Neobs study were enrolled from type 3 neonatal intensive care units, which may have created a selection bias towards those with worse RD and could limit the generalisability of our results to wider clinical practice [5]. Furthermore, there is likely to be confounding bias, whereby the indication for treatment (i.e., NIV/invasive ventilation and/or surfactant) has an impact on clinical outcome, irrespective of treatments given. For example, a lower proportion of preterm infants born at 34–36 wks versus 30–33 wks with stable RD received NIV in the delivery room, but this may reflect the better clinical condition of these infants compared with those with worsening or initially severe RD and, therefore, a greater likelihood of clinical stability over time. Conversely, infants with worsening RD might be more likely to be considered for NIV. These indication biases are very difficult to control for but have the potential to substantially influence clinical trajectories and study findings. Finally, infants were treated by physicians at each study centre according to their own clinical practice, which may be considered a strength of the study; however, the impact of local guidelines or preferences was unknown. Nevertheless, the detailed description of clinical trajectories in moderate-to-late preterm infants based on gestational age (30–33 or 34–36 wks) and clinical course (stable, worsening, or initially severe RD) in the hours and days after birth is of clinical value because there is a lack of data describing the type and duration of respiratory support given to preterm infants born after 30 wks.

From a clinical perspective, the ideal approach for RD management would be to determine factors that identify high-risk preterm infants early, and provide guidance for their care in terms of ventilatory support and surfactant therapy [19]. The validation and routine use of non-invasive techniques to guide surfactant administration in preterm infants, such as lung ultrasound [20], may also be important. These steps would ensure appropriate allocation of resources and allow for personalised care that optimises outcomes for each patient. Better understanding of clinical trajectories, as described in this analysis, is an early step towards this important goal.

In summary, our study has demonstrated that there was no significant difference in the evolution of respiratory distress according to term, and in particular, in the proportion of worsening in this population. However, the management of respiratory distress differs with late preterm infants being managed later in life and moderate premature infants weaned from ventilation at a later stage. Moreover, surfactant instillation was regularly sub-optimal, with some dosages lower from recommendations. These observations demonstrate the need to establish recommendations along the lines of those for premature infants, in order to standardize and improve respiratory distress management in moderate and late preterm infants.

## Conclusions

Clinical trajectories and management strategies for moderate-to-late preterm infants with RD differed depending on gestational age and clinical course. These observations call for a review of protocols for managing these patients and demonstrate the need to establish recommendations along the lines of those for premature infants, in order to standardize practices and improve them.

## Data Availability

Data can been asked at Chiesi SAS.

## Abbreviation

*CI*: Confidence interval
*CNIL*: Commission Nationale de l’Informatique et des Libertés
*CPAP*: Continuous positive airway pressure
*CPP*: Comités de Protection des Personnes
*FiO*_*2*_: Fraction of inspired oxygen
*HFNC*: High-flow nasal cannula
*IV*: Invasive ventilation
*NIPPV*: Nasal intermittent positive pressure ventilation
*NIV*: Non-invasive ventilation
*Q*: Quartile
*RD*: Respiratory distress
*SD*: Standard deviation
*wks*: Weeks of gestation
